# Electrophysiological signatures of dedifferentiation differ between fit and less fit older adults

**DOI:** 10.1007/s11571-020-09656-9

**Published:** 2021-02-06

**Authors:** Christian Goelz, Karin Mora, Julia Kristin Stroehlein, Franziska Katharina Haase, Michael Dellnitz, Claus Reinsberger, Solveig Vieluf

**Affiliations:** 1 Institute of Sports Medicine, Paderborn University, Warburger Str. 100, 33098 Paderborn, Germany; 2 Department of Mathematics, Paderborn University, Paderborn, Germany

**Keywords:** Spatio-temporal coherent patterns, Electroencephalography, Dynamic mode decomposition, Cardiorespiratory fitness, Older adults

## Abstract

Cardiorespiratory fitness was found to influence age-related changes of resting state brain network organization. However, the influence on dedifferentiated involvement of wider and more unspecialized brain regions during task completion is barely understood. We analyzed EEG data recorded during rest and different tasks (sensory, motor, cognitive) with dynamic mode decomposition, which accounts for topological characteristics as well as temporal dynamics of brain networks. As a main feature the dominant spatio-temporal EEG pattern was extracted in multiple frequency bands per participant. To deduce a pattern’s stability, we calculated its proportion of total variance among all activation patterns over time for each task. By comparing fit (N = 15) and less fit older adults (N = 16) characterized by their performance on a 6-min walking test, we found signs of a lower task specificity of the obtained network features for the less fit compared to the fit group. This was indicated by fewer significant differences between tasks in the theta and high beta frequency band in the less fit group. Repeated measures ANOVA revealed that a significantly lower proportion of total variance can be explained by the main pattern in high beta frequency range for the less fit compared to the fit group [F(1,29) = 12.572, *p* = .001, partial η^2^ = .300]. Our results indicate that the dedifferentiation in task-related brain activation is lower in fit compared to less fit older adults. Thus, our study supports the idea that cardiorespiratory fitness influences task-related brain network organization in different task domains.

**Supplementary information:**

The online version of this article (10.1007/s11571-020-09656-9) contains supplementary material, which is available to authorized users.

## Introduction

Age-related changes in brain network activity are characterized by dedifferentiated and compensatory involvement of wider and more unspecialized brain regions during task completion which relates to a decline of sensory, motor and cognitive skills (Baltes and Lindenberger 1997; Park et al. 2004; Sala-Llonch et al. 2015). Despite the general tendency towards a decline, brain activity and network interaction in older adults were shown to be highly dependent on the individual’s lifestyle (Smith and Thelen 2003). One influencing factor might be cardiorespiratory fitness, which has the potential to diminish the described age effects in resting state networks (Voss et al. 2016). Here, we aimed to detect characteristics of brain network activity representing rest- and task-related specificity of information processing in elderly with different cardiorespiratory fitness levels.

Dedifferentiation was detected in task-related fMRI studies investigating reduced neural specialization as well as compensatory involvement of task relevant brain areas in older adults, which is considered part of dedifferentiation in this work (Sala-Llonch et al. 2015). Furthermore, it was shown that regional hyperactivation in older compared to younger participants was related to changes in both fMRI- and EEG-derived task-related functional networks (Geerligs et al. 2012, 2014). These changes also showed up across different tasks leading to a less task-specific activation of task-related brain network processes (Dennis and Cabeza 2011). Moreover, age-related changes seem to be multidimensional and complex. Chen et al. (2017) and Nobukawa et al. (2019) found age-related reorganization in the dynamics of brain networks, indicated by either higher complexity or irregularity in brain network patterns (Nobukawa et al. 2019) or changes in variations of functional connectivity over time (Chen et al. 2017). These findings might relate to increased background activity or neural noise throughout task execution (Hong and Rebec 2012). Increases in neuronal noise have been suggested as contributing factors to cognitive deficits and have been linked to neurobiological mechanisms (such as a decline in the dopaminergic neuromodulation) associated with a decrease in the distinction of cortical representations due to dedifferentiated brain activation (Li et al. 2001; Sala-Llonch et al. 2015).

Douw et al. (2014) related a higher interconnected functional modular topology of MEG derived resting state brain networks to cardiorespiratory fitness in middle aged participants. The findings indicate that cardiorespiratory fitness has an influence on brain networks and might bear the potential to counteract age-related changes, i.e., dedifferentiation and compensatory mechanisms. Indeed, Stillman et al. (2019) reviewed effects of physical activity and fitness on fMRI derived resting state brain networks in older adults and reported opposing effects on age-related changes. In this context, several authors reported an increase of connectivity within resting state networks in older adults due to cardiorespiratory fitness, which suggests less dedifferentiated brain functioning (Prakash et al. 2011; Voss et al. 2010, 2016). However, less is known about the influence of cardiorespiratory fitness on task-related brain network processes and dedifferentiation across different tasks in older adults. The influence of cardiovascular fitness on the decrease in specificity of information processing in the sensory, motor, and cognitive areas, in which age-related decline is reported, seems to be particularly relevant for everyday life and is yet unclear. Especially the investigation of dynamic processes of central information processing could provide new insights into this impact.

EEG allows to study brain dynamics with high temporal resolution capture changes of the temporal characteristics of functional networks. Due to the high complexity of age-related reorganization of brain activity and its interaction with cardiorespiratory fitness, we chose a holistic approach which combines the representation of spatial and temporal brain activity patterns. Dynamic mode decomposition (DMD) is an algorithm that was developed in the field of fluid dynamics (Rowley et al. 2009; Schmid and Sesterhenn 2008). It was recently applied to various other fields, including neuroscientific data (Brunton et al. 2016; Casorso et al. 2019; Gölz et al. 2018; Kunert-Graf et al. 2019; Vieluf et al. 2018). DMD combines the properties of spatial and temporal decomposition methods enlarging classical functional connectivity (FC) approaches based on bivariate connectedness between voxels or electrodes, which is frequently done in literature (Sporns 2013).

Utilizing DMD, we therefore aimed to study the influence of cardiorespiratory fitness on task-related brain network activity by assessing coherent spatio-temporal patterns of EEG in rest as well as tasks representing the sensory, motor, and cognitive domains, respectively. Albeit exploratory, we expected electrophysiological signatures of dedifferentiation, i.e., less task specificity in different tasks in fit compared to less fit individuals. In addition, we hypothesized that fit older adults have lower levels of neural noise, which translates into a higher prominence of dominant brain network patterns.

## Materials and methods

The data were collected during an intervention study, which was registered at the German Clinical Trials Register (DRKS00014921) and took place at Paderborn University. The study protocol was approved by the ethics committee of the University of Muenster. Written informed consent to participate in the study was obtained by each participant before the experiments. No compensation was offered.

### Participants

Participants were recruited via local newspaper and social media advertisements as well as by personal contact with organizations providing leisure activities for seniors. Participants were included if they (1) were above 60 years old, (2) free of diagnosed neurological or mental diseases and (3) right-handed. Half of the participants participated in a golf training and half continued their normal activities prior to the recording. In the context of this study golf was considered as part of the daily activities. All included participants reported subjective memory complaints in daily life but had no diagnosed form of dementia or its preclinical manifestation mild cognitive impairment, and were therefore considered healthy. All participants scored below 13 on a neuropsychological test battery (see Alzheimer’s Disease Assessment Scale-Cognitive Subscale (ADAS-cog) Table 1). In sum, a total of 41 participants between 60 and 77 years of age participated in this study (age: 67 ± 4.16, gender: 22 ♀, 19 ♂; see Table 1). All participants had normal or corrected to normal vision.

### Screening

In preceding appointments, participants’ cardiorespiratory fitness was assessed with a 6 min walking test (Enright 2003). Participants had to walk 6 min as far as possible with a fast and constant pace. The distance was assessed as marker of cardiorespiratory fitness as this test was shown to be a reliable and valid way to test physical endurance in older adults (Rikli and Jones 1998; Zhang et al. 2017). Prior to the EEG measurement and in relation to the domains of the main tasks, maximum voluntary contraction (MVC) as well as reaction time (RT) and tactile threshold (TT) were assessed. MVC measurements consisted of three 5 s lasting maximum precision grip trials with 60 s rest in between (Vieluf et al. 2013). RT was assessed with auditive stimuli, i.e., 60 spoken letters presented via speakers. As soon as a letter was presented, participants had to press a foot switch immediately with the right foot. The foot switch was positioned in a standardized position next to the right foot. Reaction time was measured in relation to stimulus onset (Voelcker-Rehage and Alberts 2007). The TT was detected on the non-dominant hand as a 2-point discrimination test. Participants were asked to distinguish between one-point and two-point stimulation. The distance between the stimulation points was successively increased by 1 mm starting at a minimum distance of 1 mm. TT was achieved when the participant could clearly distinguish 7 out of 10 stimulations presented (Finnell et al. 2004).

### EEG experiments

All EEG measurements were recorded with an actiCap electrode cap and BrainAmp standard amplifiers (Brain Products, Munich, Germany). We recorded brain activity at 128 electrodes with a sampling rate of 500 Hz. Ground and reference electrodes were fixed at FPz and FCz, respectively. Impedances were kept below 15 kΩ.

At first, EEG was recorded four minutes in a rest condition in supine position with eyes closed. During the tasks, participants sat 80 cm in front of a screen (23′′, 1920 × 1080 pixels at 60 Hz, AOC, Taipei, Taiwan). Their right arm rested comfortably on an armrest grasping a force transducer with index finger and thumb in precision grip (1022-C3-20 kg, SOEMER, Lennestadt-Elspe, Germany). Participants placed their left index finger on a braille device (P11, Metec Ag, Stuttgart Germany). In addition, speakers were placed approximately 50 cm behind the participants as well as a footswitch (StealthSwitch SS1R4 Pro USB, StealthSwitch, Highwood, IL, United States) next to their right foot. The tasks consisted of a motor task (force control), a cognitive task (auditive 2 back), and a sensory task (sensory oddball task) lasting 90 s. Each task was presented 2 times.

#### Motor task

**Fig. 1:**
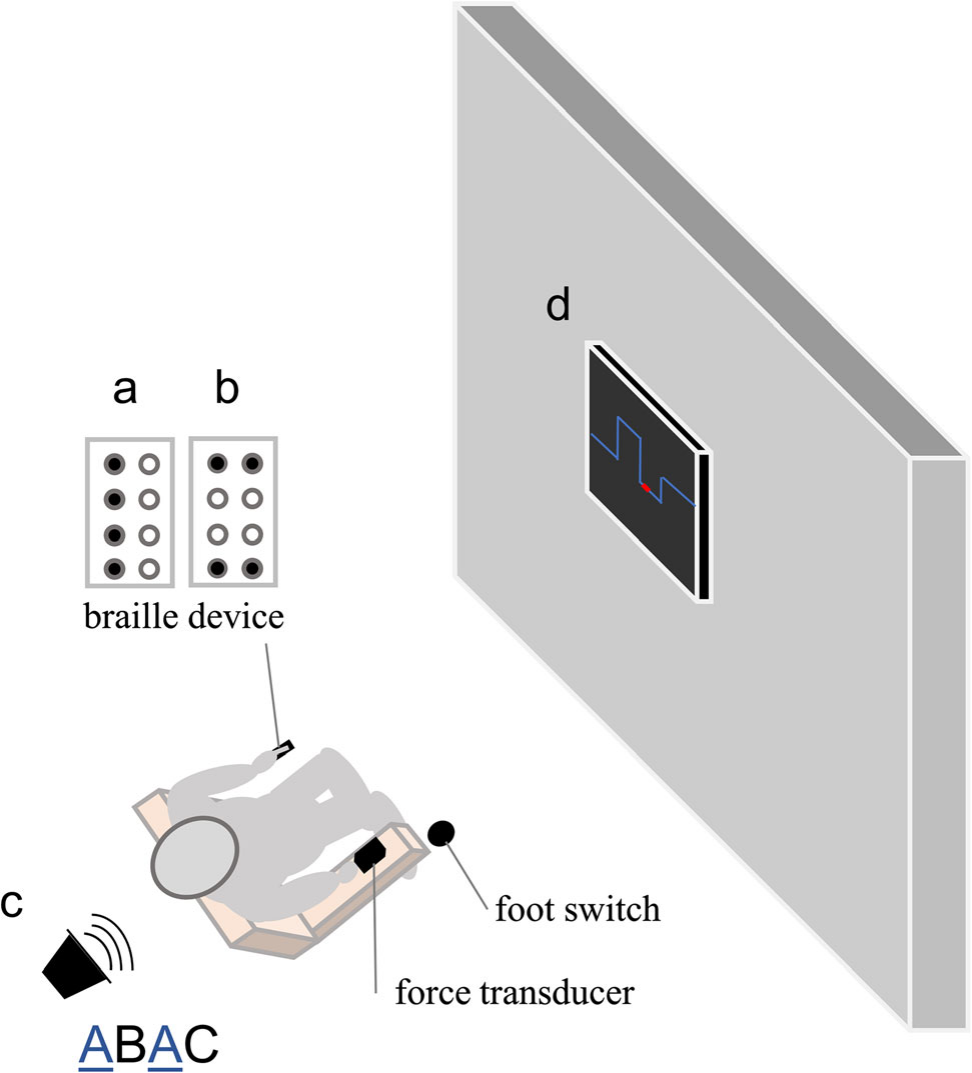
Experimental setup of the sensory (**a**, **b**), n-back (**c**), and motor task (**d**)

Participants had to apply force to a force transducer with their index finger and thumb in precision grip to match a visually presented target (Voelcker-Rehage et al. 2006). The visual target was a line that moved from the right to the left on the screen for 90 s and changed level every 3 s in randomized order between heights that represents 10%, 20% and 30% of their individual MVC (see Fig. 1). Mean force level was set to 20% of participants MVC throughout the experiment. Participants were given online feedback on a screen 80 cm in front of them. Target line was displayed in blue whereas a red curser represented the applied force (see Fig. 1d).

#### Cognitive task

Participants were asked to listen to a sequence of letters presented via two speakers behind them and press the foot switch with the right foot, if a letter appeared again two letters later (2-back; see Fig. 1c). The sequences consisted of 60 letters in predefined randomized selections with 20% matching rate (Bopp and Verhaeghen 2018; Gajewski and Falkenstein 2014).

#### Sensory task

The braille device presented two stimuli (see Fig. 1a and 1b) to the participants with a randomized inter stimulus interval of 0.8–1.2 s (mean 1 s) and a duration of 0.5 s each. In this passive oddball design no response was required from the participants. Pattern (a) was set to 80% occurrence and pattern (b) to 20% (Reuter et al. 2012, 2013, 2014).

### Data analysis

Data analysis was performed with BrainVision Analyzer 2.1.2 (Brain Products, Munich, Germany), MATLAB 2018b (Mathworks, Natick, MA, United States), EEGLAB 14.1.2 (Brunner et al. 2013), as well as Brainstorm 3.4 (Tadel et al. 2011) software packages. EEG data were cut from task onset to offset. Next, data were filtered with a zero-phase shift 4th order Butterworth filter from 1 to 30 Hz and an additional notch filter at 50 Hz implemented in Brain Vision Analyzer 2.1.2 was applied. After down sampling to 200 Hz, data were inspected for bad channels as well as electrode bridges using Alschuler’s et al. (2014) algorithm, which is based on the electrical distance of neighboring electrodes. To avoid false positives, coherence between adjacent channels was additionally checked for each pair of channels, where a value of 1 indicates complete coherence and a value of 0 indicates complete incoherence. Bad channels or channel pairs identified by Alschuler’s et al. (2014) algorithm and with a coherence above 0.97 were rejected. Participants whose rejection rate was above 15% or exhibited a bridged reference were excluded from further analysis. Seven participants had to be excluded here. From the remaining participants, we excluded on average 4% of the electrodes per participant. Then, data was re-referenced to common average and segmented into non-overlapping segments of 150 data points (0.75 s). Before performing an ICA on the re-concatenated data in EEGlab (AMICA, Palmer et al. 2011) for artifact rejection, segments containing non stereotypical artifacts identified by visual inspection were excluded. ICA components reflecting unambiguous artifacts such as eye blinks, heartbeat or muscle activity were marked as bad (mean rejection rate: 19 ± 5%) and excluded. After reconstruction of the signals all rejected channels were interpolated using spherical spline interpolation (Perrin et al. 1989) and checked again visually for segments containing artifacts.

In order to extract spatio-temporal coherent patterns within the EEG we decomposed the preprocessed data segments with the exact DMD algorithm described in Brunton et al. (2016) after Tu et al. (2014). Specifically, we constructed the two data matrices $X \in {\mathbb{R}}^{{n \times \left( {m - 1} \right)}}$ and $X^{{\prime }} \in {\mathbb{R}}^{{n \times \left( {m - 1} \right)}}$, where $X$ is the original data matrix and $X^{{\prime }}$ is the data matrix shifted by one time point containing $m - 1$ datapoints of $n$ electrodes. Moreover, the relationship between these two matrices can be expressed with a linear operator $A$ describing the underlying dynamical process such that

$$X^{{\prime }} = AX.$$

The DMD of $X$ is then obtained via eigen-decomposition of $A$. In other words by analyzing the relationship between $X$ and $X^{{\prime }}$ in a given time window (see Brunton et al. 2016 for details of the algorithm) it is possible to approximate the linked spatial and temporal characteristics. Therefore, we can obtain an approximation $\hat{X}$ of the observed measurement $X$ by defining a dynamical model

$$\hat{X} = \varPhi \exp \left( {\varOmega t} \right)z.$$

The matrix $\varPhi$ contains the dynamic modes, i.e., eigenvectors of $A$ (see Theorem 1 in Tu et al. 2014) and $\varOmega = \frac{\log \left( \varLambda \right)}{\Delta t}$ reveals the dynamics of the system, where the diagonal matrix $\varLambda$ contains the DMD eigenvalues, i.e., eigenvalues of $A$, on its diagonal. The variable $t$ denotes time whereby $\Delta t = 0.005\;{\text{s}}$. The constant $z$ can be obtained from the initial conditions $x_{1} = \varPhi z.$ Moreover, it is possible to obtain the oscillation frequencies in cycles per second (Hz) from $\varOmega$:

$$f = \frac{imag\left( \varOmega \right)}{2\pi }.$$

We conjecture that the dimension of the underlying system is larger than the one obtainable from the original data matrix $X$. Thus to avoid an underestimation, we propose to increase the dimension of the data matrix via the delay embedding method applied by Tu et al. (2014) and described in Brunton et al. (2016) as well as in Cohen (2018). In order to estimate the optimal values of the stacking factor *h* and the window size *w* we applied an error analysis on participant data of five randomly selected participants as described in Brunton et al. (2016). Based on these results we decided to choose a stacking depth of *h* = 5 and *w* = 150 data points.

To obtain the influence of each electrode in the DMD mode, the absolute DMD values associated with the θ (4 to < 7 Hz), α (7 to < 12 Hz), low β (12 to < 16 Hz) and high β (16 to < 30 Hz) frequencies were determined obtaining DMD mode magnitudes. Each DMD mode reflects a spatially coherent structure associated with a certain dynamic behavior, i.e., growth or decay and oscillation, where magnitude indicates the element’s (electrode) involvement in the mode. Next, we calculated a singular value decomposition (SVD) over all frequency specific modes over all time windows per participant to reduce the dimension of all time windows to its main features. Besides we calculated the proportion of variance among all activation patterns over time for each SVD mode:

$$R^{2} = \frac{{s_{i}^{2} }}{{\mathop \sum \nolimits_{j} s_{j}^{2} }} \times 100,$$

where $s_{j}$ are the singular values obtained via SVD and $s_{i}$ denotes the $i$-th singular value corresponding to the $i$-th SVD mode. Since the first SVD mode explained most of the variance of the activation patterns in all subjects (M = 81.7% ± 1.1%) we extracted only the first mode to obtain the dominant feature of all DMD modes (DMD main mode) in all analyzed time windows. Moreover, we calculated the proportion of variance among all activation patterns explained by the obtained DMD main mode. The higher the proportion the more variation among all activation patterns during task completion is captured by this dominant mode which is considered representative for stability or prominence of this pattern.

As DMD is closely related to source separation, we used sLoreta to visualize the obtained spatial DMD maps in source space to demonstrate its relation. We created a three layer Boundary Element (BEM) forward model using the brainstorm toolbox based on a template of the McConnell Brain Imaging Center (MNI/ICBM152) and fitted the electrodes using recorded individual electrode positions per subject (Fonov et al. 2009; Gramfort et al. 2010; Pascual-Marqui 2002; Tadel et al. 2011).

### Statistical analysis

All statistical analyses were conducted using SPSS 24 for Windows (IBM, Armonk, NY, United States), Matlab 2018b (Mathworks, Natick, MA, United States) with the additional Brainstorm package (Tadel et al. 2011) as well as R with RStudio (version 1.1.456) and the additional cramer-package (Baringhaus and Franz, 2004). To test differences between fit and less fit participants we divided them based on their performance in the 6-min walking test with a median split for men and women separately to account for gender differences (Bohannon 2007) in two groups. In order to characterize brain network activity and test for task specificity of the obtained DMD maps we compared the task- and rest-specific DMD mode maps pairwise with a permutation *t*-test for dependent measurements. The permutation approach was chosen as a non-parametric alternative and offers the advantage of calculating the exact test statistic. For this we chose a Monte Carlo approach with 10,000 randomizations, i.e., all possible values of the test statistics have been determined with a 10,000-fold random reordering of the data, the distribution of the t-test statistics under the null hypothesis (Maris 2012; Maris and Oostenveld 2007). By choosing this nonparametric statistic, we intended to account for the high dimensionality of the EEG data causing possible inaccuracies in test assumption requirements. By using an exact test statistics we intended a reduction of type I and type II errors (Maris and Oostenveld 2007). All p values were corrected with false discovery rate to account for type I errors (Benjamini and Hochberg 1995). For a statistical evaluation of the group differences, we exploratively compared the multivariate distribution of obtained *t*-values in each frequency band with Cramér tests between the groups. This nonparametric two sample test works on Euclidean interpoint distances to test the equality of the underlying distributions (Baringhaus and Franz, 2004).

We further analyzed the variance of all DMD modes explained by the DMD main mode with repeated measurement analysis of variance (ANOVA) with the between factor group (2; fit, less fit) and the within factor task (4; rest, motor, cognitive, sensory) for each frequency band (θ, α, low β, high β). Shapiro–Wilk and Box tests showed no violation of normal distribution and homogeneity of error variances (all *p* > .05). There was homogeneity of the error variances, as assessed by Levene’s test except for the variables in the theta and alpha band during n-back and sensory tasks. We nevertheless report those values, as ANOVA was shown to be a robust test statistics in groups with almost the same size larger than 10 (Box 1954; Schmider et al. 2010). Significant interactions and main effects were followed by Bonferroni corrected pairwise comparisons. Independent t-tests and Mann–Whitney *U* tests, in case of violation of normal distribution, were used to check for differences in characteristics between the groups. The level of significance was set a priori to α = 0.05 for all tests.

## Results

The personal characteristics of the final sub-sample are shown in Table 1. Mann–Whitney *U* tests and independent *t*-tests showed no significant differences between the groups of fit and less fit participants for personal characteristics or screening variables, except for the walked distance in the six minute walking test (fit: M = 765 m, SD = 127 m; less fit: M = 549 m, SD = 100 m, t(29) = − 5.25, *p* < .001).

**Table 1:** Comparison of characteristics of the fit and less fit group

Parameter	Less fit N = 16	Fit N = 15	Statistical value	*p*-value
Sex	8♀, 8♂	8♀, 7♂		
Age (years)	M = 68.56, SD = 4.13	M = 66.26, SD = 3.67	*t* = 1.637	.114
Height (cm)	M = 173.75, SD = 10.93	M = 171.93, SD = 10.02	*t* = 0.481	.634
Weight (kg)	M = 78.38, SD = 15.06	M = 79.60, SD = 10.76	*t* = − .259	.898
ADAS-Cog	M = 7.13, SD = 2.5	M = 5.87, SD = 3.14	*t* = 1.230	.225
Tactile Threshold (mm)	Median = 3, SD = 0.72	Median = 3, SD = 0.70	*U* = 103	.459
RT (s)	M = 1.03, SD = 0.29	M = 0.91, SD = 0.19	*t* = 1.290	.207
MVC (N)	M = 53.91, SD = 18.30	M = 62.32, SD = 21.45	*t* = − 1.177	.249
PASE score	Median = 144.9, SD = 46.75	Median = 161.35, SD = 50.40	*U* = 93	.600
Six-minute walking (m)	M = 563.29, SD = 115.6	M = 750.21, SD = 138.5	*t* = − 5.249	.000*
	(norm: ♀ ≥ 60 years: 475 (95%-CI [448, 503])/♂ ≥ 60 years: 560 (95%-CI [511, 609])			

*Indicates a significant difference. Norm values of six-minute walking test from Bohannon (2007). *MVC* Maximum voluntary contraction, *ADAS*-*cog* Alzheimer’s disease assessment scale-cognitive subscale, *PASE* physical activity scale for the elderly

### DMD mode maps

**Fig. 2:**
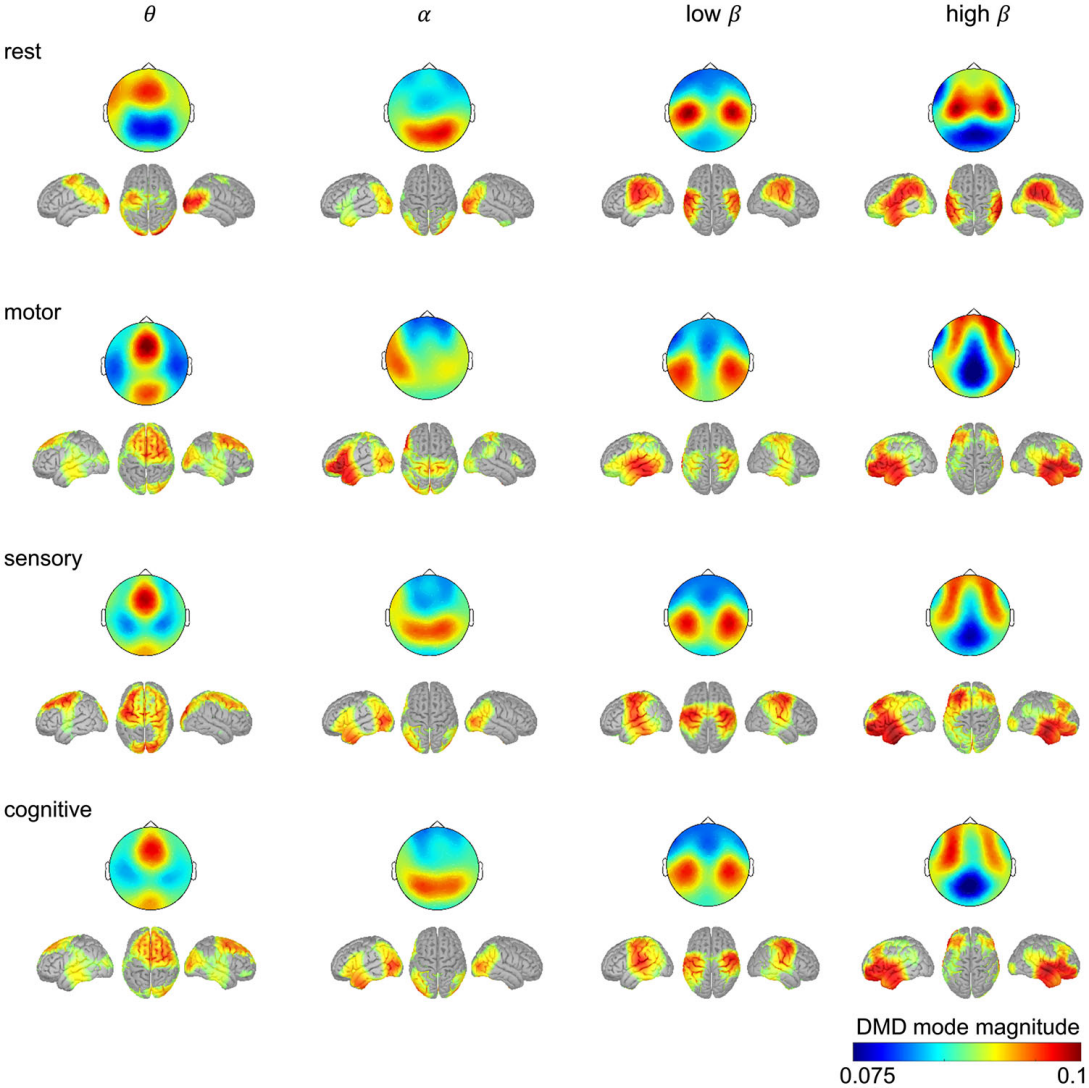
DMD main mode features during rest with eyes closed and the three different task conditions (motor, sensory, cognitive) in the frequency ranges of θ (4 to < 7 Hz), α (7 to < 12 Hz), low (12 to < 16 Hz) and high β (16 to < 30 Hz) as well as their source representation. Each row represents a condition and each column represents a frequency band, thus there are four topographic maps per condition. Maps represent the mean over all participants

Figure 2 illustrates the DMD topographical maps with an estimated source localization. Note that the source localization is only presented for visualization purposes here. Each DMD mode reflects a pattern of correlation in space at certain frequencies (Kutz et al. 2016). Low values indicate low contribution to the mode whereas high values indicate high contribution to that mode. Noticeable DMD main mode maps at rest showed frequency specific characteristics. The θ activity was dominant over frontal-central areas and showed highest values in the motor task. Dominant α activity over occipital regions during rest was suppressed and shifted to temporo-parietal areas. The low β activation showed the highest values at rest above the central areas and became more extensive in temporal direction during tasks. While the high β-band showed a similar central activation during rest, the activity in this frequency band changed towards a dominant activation over frontal with a shift towards centro-temporal areas that seemed to be most pronounced during the motor task (see Fig. 2).

**Fig. 3:**
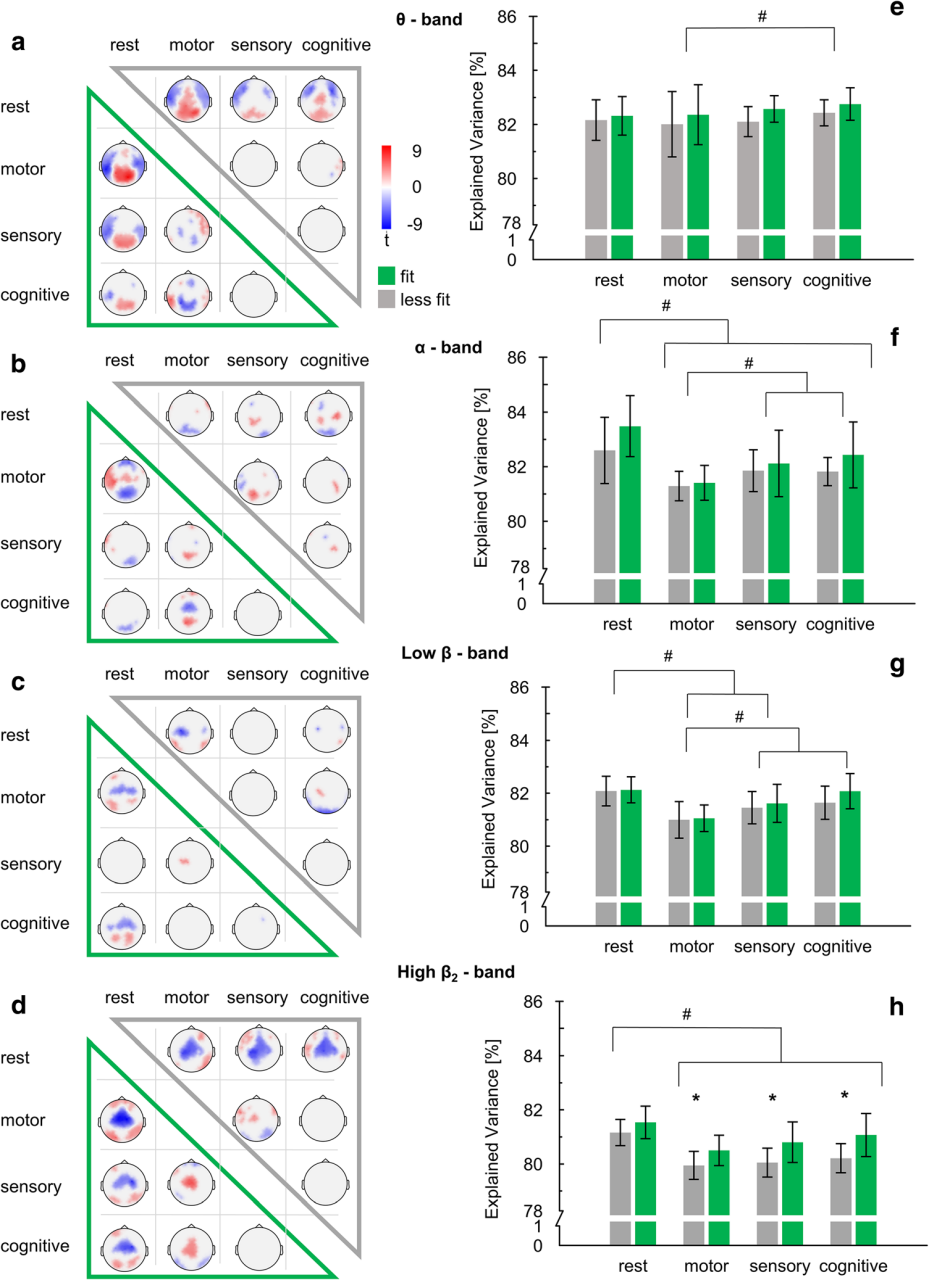
**a**–**d** Statistical *t*-maps of significant differences of DMD main mode features in θ, α, low- and high β between the conditions divided in fit (green) and less fit (grey) participants. Only *t*-values with corresponding corrected *p* value < .05 are visualized. The opposite side of each group served as second term in the *t*-test for each group. **e**–**h** DMD main mode goodness of fit expressed in % of variance they explain as group mean and standard deviation. * indicates significant pairwise comparisons of the main effect group. # indicates significant pairwise comparisons of the main effect task

Figure 3a–d illustrates the comparisons between the tasks for the fit and less fit groups separately in the analyzed frequency bands (θ, α, low β and high β) as topographic *t*-maps. For simplicity and clarity of presentation only minimum and maximum *t*- and corresponding *p*-values as well as their electrode position are presented in Tables 2 and 3. Means and standard deviations per electrode are available in the supplementary material (Online Resource 1).

**Table 2:** Minimum and maximum *t*-values as well as corresponding *p*-value and their electrode position (loc.) of the group specific comparison between task conditions and resting state condition

vs.	Rest											
	Fit						Less fit					
	*t*_min_	*p*	Loc.	*t*_max_	*p*	Loc.	*t*_min_	*p*	Loc.	*t*_max_	*p*	Loc.
	θ											
Motor	− 8.64	< .001*	TP7	9.32	< .001*	PO4	− 6.52	.005*	FTT9h	7.32	.005*	PPO6h
Sensory	− 5.65	< .001*	TP7	5.34	< .001*	POO1	− 5.57	.009*	F5	3.79	.024*	PPO6h
Cognitive	− 5.70	.01*	CP5	4.58	.01*	PPO6h	− 8.23	.006*	FFC6h	4.19	.007*	Cz
	α											
Motor	− 6.82	.003*	AFp2	6.56	.003*	CCP5h	− 4.25	.002*	PO3	5.00	.020*	F10
Sensory	− 4.67	.038*	O2	5.49	.038*	FT9	− 4.29	.023*	O2	4.43	.024*	FT9
Cognitive	− 4.12	.050*	POO1	4.24	.047*	FT9	− 4.61	.009*	O2	5.92	.009*	FT9
	Low β											
Motor	− 5.10	.021*	C4	3.99	.030*	AFF5h	− 7.44	.006*	C3	4.92	.009*	P7
Sensory	− 5.83	.003*	T7	5.80	.003*	PO8	− 3.57	.129	C3	4.05	.129	P7
Cognitive	− 4.61	.013*	FCC4h	4.07	.013*	P4	− 5.49	.003*	C3	4.37	.030*	P8
	High β											
Motor	− 9.73	.009*	C2	6.07	.003*	O1	− 7.09	.003*	CCP3h	4.80	.003*	PPO10h
Sensory	− 7.90	.010*	FCC4h	3.61	.017*	PO8	− 7.62	.003*	CP3	3.62	.013*	AF7
Cognitive	− 7.73	.002*	FCC4h	4.83	< .001*	PPO6h	− 6 − 57	.003*	CCP1h	4.95	.003*	TP8

*Indicates a statistically significant difference

**Table 3:** Minimum and maximum *t*-values as well as corresponding *p*-value and their electrode position (loc.) of the group specific comparison between all task conditions

vs.	Motor											
	Fit						Less fit					
	*t*_min_	*p*	Loc.	*t*_max_	*p*	Loc.	*t*_min_	*p*	Loc.	*t*_max_	*p*	Loc.
	θ											
Sensory	− 3.29	.032*	PO4	4.69	.031*	FFT10h	− 3.36	.181	PPO6h	3.42	.181	T8
Cognitive	− 7.48	.002*	P3	6.63	.002*	TP7	− 4.13	.029*	P6	4.16	.029*	TP10
	α											
Sensory	− 426	.006*	P9	5.25	.006*	PPO1h	− 4.44	.018	TTP7h	5.92	.018*	PPO1h
Cognitive	− 5.49	.006*	FCz	6.84	.006*	PPO1h	− 4.31	.031*	F10	4.98	.029*	CPP6h
	Low β											
Sensory	− 3.12	.131	AF3	4.61	.043*	C1	− 3.61	.100	P9	4.03	.100	F9
Cognitive	− 3.10	.240	Fp2	3.76	.24	CP1	− 8.02	.004*	O9	4.43	.016*	CPP6h
	High β											
Sensory	− 4.15	.013*	AFp2	6.10	.003*	Cz	− 3.81	.037*	PPO10h	6.41	.013*	FT7
Cognitive	− 4.13	.014*	PPO9h	4.50	.014*	TP7	− 4.68	.070	Iz	3.87	.070	F10

vs.	Sensory											
	Fit						Less fit					
	*t*_min_	*p*	Loc.	*t*_max_	*p*	Loc.	*t*_min_	*p*	Loc.	*t*_max_	*p*	Loc.
	θ											
Cognitive	− 2.93	.292	FFC6h	3.11	.292	T7	− 3.66	.146	TP10	3.65	.146	FC4
	α											
Cognitive	− 3.08	.606	FFC4h	2.33	.292	F9	− 4.05	.013	FFC1h	4.23	.013*	CCP4h
	Low β											
Cognitive	− 3.61	.039*	FFC4h	3.16	.221	PO3	− 2.72	.155	Iz	3.47	.155	P8
	High β											
Cognitive	− 3.73	.103	Cz	3.42	.103	PPO1h	− 3.28	.219	C1	2.36	.44	AF8

*Indicates a statistically significant difference

For both groups and frequency bands, DMD main mode maps differed between each task and rest with the strongest differences in the θ-band and in the high β-band. Both groups θ-band main mode during task was characterized by higher activity over parieto-occipital areas and lower activity over fronto-temporal areas compared to rest. In contrast, in the high β-band, the differences between task execution and rest are marked by lower activation over (parieto-) central and higher activation over frontal and occipital areas in both groups.

Comparing the *t*-distributions with Cramér tests between the two groups revealed significant differences for all frequency bands (all *p* < .001). Studying the *t*-maps in group comparison, finer spatial differences can be observed. The differences between task and rest seem to be similar between the groups in the θ-band and in the high β-band. However, the differences between tasks and rest might be stronger in α- and low β-bands in the fit compared to the less fit group. This is especially observed in the α-band with lower parieto-occipital/frontal and higher temporal-central activation during motor task compared to the rest condition. Similar trends could be observed in the low β-band with higher parieto-occipital and lower central activation in the cognitive task compared to (see Fig. 3; Table 2).

There were likewise fewer pronounced differences between the task conditions with the highest expression between the motor task and the two other tasks in both groups. The differences, however, seemed to predominate in the fit group, especially in the θ-band and in the high β-band in the motor task compared to the sensory and cognitive tasks. In the fit group the θ-band is characterized by reduced activation of parieto-occipital and higher activation of temporo-parietal areas in the n-back task as well as higher temporo-frontal and a reduced activation of central and parietal areas of the sensory compared to the motor task. High β-band differences were present in form of activation over central areas and sporadically decreased over frontal or occipital areas of the cognitive and sensory tasks compared to the motor task. There were likewise only small differences in both groups in task comparisons in the low β-band. Comparison of cognitive and sensory task revealed almost no differences in both groups (see Table 3).

### Explained variance

Results of DMD main mode explained variance are illustrated in Fig. 3e–h. Repeated measures ANOVA revealed a significant effect of task in the θ-band [F(3, 87) = 26.704, *p* < .001, partial η^2^ = .479] but none for group [F(1, 29) = 3.174, *p* = .085, partial η^2^ = .099]. Post hoc tests revealed significant differences between the motor and the n-back task independent of group (*p* = .023).

Moreover, there was a significant main effect of task in the α-band [F(3, 87) = 4647, *p* = .005, partial η^2^ = .138] but no significant effect of group [F(1, 29) = 2.79, *p* = .106, partial η^2^ = .088]. All tasks differed significantly from rest (sensory: *p* < .001; motor: *p* < .001; cognitive: *p* = .002). Sensory and motor differed significantly (*p* = .008) as well as motor and n-back (*p* < .001).

In the low β-band there was a significant effect of task [F(3, 87) = 31.399, *p* < .001, partial η^2^ = .520], but neither an effect of group [F(1, 29) = 1.034, *p* = .318, partial η^2^ = .030] nor an interaction between task and group [F(3, 87) = 1.204, *p* = .313, partial η^2^ = .040] were significant. The sensory task and motor task explained variance showed significantly lower values compared to rest (*p* < .001). Motor task was characterized by significantly lower values compared to the sensory task (*p* < .001) and cognitive task (*p* = .001) as well as higher values of the cognitive compared to the sensory task (*p* = .020).

Repeated measures ANOVA revealed a significant main effect of task [F(3, 87) = 41.855, *p* < .001, partial η^2^ = .600] and group [F(1, 29) = 12.572, *p* = .001, partial η^2^ = .300] in the high β-band. There was no significant interaction of task and group [F(3, 87) = 2.004, *p* = .119, partial η^2^ = .070]. Bonferroni-corrected post hoc tests demonstrated significantly higher values in rest compared to all tasks (all *p* < .001).

## Discussion

Utilizing the multivariate analysis method dynamic mode decomposition (DMD), we aimed to find characteristics of task specific brain network patterns and their differences in fit compared to less fit elderly. We analyzed EEG data recorded during sensory, motor, and cognitive tasks in comparison to rest. DMD revealed frequency-dependent spatio-temporal scalp patterns that appear at rest and three different tasks. In fit older adults, these patterns showed trends of higher task specificity pointing to less dedifferentiated brain activity. Furthermore, we found this pattern’s proportion of total variance explained higher in fit participants which might indicate less neural noise during task execution. The results jointly support the idea that physical fitness reduces the impact of dedifferentiation.

We identified main features of frequency specific brain network dynamics expressed in task-specific EEG patterns. As DMD can be conceptualized as a combination of well-known spectral analysis methods like the Fourier Transform with the Fast Fourier Transform algorithm (FFT) and spatial decomposition with principle component analysis (PCA) our results are comparable to well-known EEG-power characteristics modified throughout the tasks reflecting markers of frequency specific network interaction and modulation dominant throughout the tasks. However, in addition DMD links the assessed spatial and temporal properties providing a low-dimensional representation of the underlying time-variable complex system.

In examining the DMD derived EEG patterns we aimed to study selectivity of neural responses, or dedifferentiation, across rest and three different tasks. The dominant fronto-central θ activation found here has been discussed as being sensitive to cognitive involvement and was dominant in the task conditions compared to rest (Jensen and Mazaheri 2010; Onton et al. 2005; Siegel et al. 2012). Occipital α activity being suppressed in the task conditions in this study has been discussed as a marker for the suppression of the visual network during visual attention. In general, the activity of α could play a role in so-called gating by inhibition processes, i.e., the selective activation of task-relevant and suppression of task-irrelevant areas and networks (Jensen and Mazaheri 2010). Moreover, high β was discussed as a marker of large-scale coupling and sensorimotor information integration showing wider distribution in the tasks compared to the rest condition (Siegel et al. 2012). Overall, the expression of these patterns was different in rest and the three tasks and can be regarded as reflection of task-specific network activation processes linked to aforementioned processes. Indeed, comparing these patterns across tasks both groups showed differences between rest and all tasks in all frequency bands, most dominant in θ- and high β-bands. The role of these two frequency bands could indicate the importance of cognitive control and large-scale coupling in maintaining functioning in older age pointing to compensatory activity as it was found by Vlahou et al. (2014) and Knyazev et al. (2015).

In line with the influence of cardiorespiratory fitness on resting state networks we hypothesized to find task specificity of these task-related brain network patterns less pronounced in less fit participants compared to fit participants. By comparing information processing between a motor, cognitive, and sensory task, we were able to study the influence of cardiorespiratory fitness on the reduction of neuronal specialization between tasks. These task domains should correspond to the domains in which age-related decline is reported and thus provide a high relevance for everyday life. In fact, the differences between the tasks seem to be more pronounced in the fit group compared to the less fit group. In other words, task-related neural responses of fit compared to less fit participants showed higher differentiation across task. This finding thus might indicate opposing effects of cardiorespiratory fitness on age-related reduction of neuronal dedifferentiation found in literature in motor, cognitive as well as visual tasks (Carp et al. 2011; Park et al. 2004; Rajah and D’Esposito 2005) and is in line with findings showing these effects on resting state networks (Voss et al. 2016). However, there were no clear significant differences between the sensory and the cognitive task in both groups. The undetectable difference indicates a similar degree of dedifferentiation that is independent of the cardiorespiratory fitness level. On the other side it has been shown that both tasks are highly dependent on the cognitive resources of working memory, this may have masked task-specific differences between the two tasks (Dehghan Nayyeri et al. 2019). It is also noteworthy that the clearest differences between the tasks were again found in the theta and high beta range. This could again show the influence of cognitive control processes and large-scale coupling in maintaining function in older adults and could also indicate that these processes are influenced by cardiorespiratory fitness.

Based on recent reports on age-related changes in the dynamics of reorganization processes linking increased levels of neural noise and dedifferentiation, we characterized each DMD main mode in terms of the proportion of the total variance of all activation patterns explained by the dominant pattern. By analyzing this as stability or prominence of the main DMD mode throughout task execution, we intended to take into account the changes in the dynamics of age-related reorganization reported in the literature (Chen et al. 2017; Li et al. 2001; Nobukawa et al. 2019). Furthermore, we aimed to investigate differences in this dynamic between subjects with different levels of cardiorespiratory fitness. Expecting lower levels of neural noise in the fit group, we found lower values in high β main mode explained variance in the less fit group independent of the task. Carefully interpreted, this could lead to a more targeted, task-specific information processing throughout task execution. With this finding, the present study can contribute to previous literature describing effects of cardiorespiratory fitness on age-related cognitive changes and brain network dynamics. To the best of our knowledge, no study has yet investigated the influence of cardiorespiratory fitness on dynamic brain network processes in older adults in the context of dedifferentiation. The comparison of brain networks during a variety of tasks and analyzing the similarities and differences allows to gain insights into functional organizations of task relevant activities. Our results point to less neural noise throughout task execution in the fit group and extend the existing knowledge about the influence of cardiorespiratory fitness on dynamic processes and age-related changes in these processes.

On a methodological level, decomposing EEG spatial temporal dynamics with DMD is dependent on delay embedding, i.e., stacking factor, as well as number of electrodes and chosen window size. We picked the parameters according to an error analyses over several participants to choose the optimal parameters. Moreover, delay embedding was used by other authors before (Brunton et al. 2016; Cohen 2017). As we measured high density EEG, we decided to check for bridging artifacts. Therefore, we used an algorithm proposed by Alschuler et al. (2014) which identifies bridges based on the electrical distance distribution of the signals. However, Alschuler et al. (2014) note that this algorithm might be too conservative. We therefore decided to double check this step and calculated the coherence between neighboring channels.

In order to achieve a high level of comparability between tasks and groups of participants, we decided to reduce dimensionality of the variables with SVD and chose the most important characteristic, i.e., the first principle component. We decided not to study further components since we describe a coarse phenomenon such as dedifferentiation.

Moreover, we used the 6-min walking test to asses cardiorespiratory fitness instead of VO_2_max measurements. This test is highly influenced by motivational aspects. However, there is a strong relation between the 6-min walking test and VO_2_max and it is used as a common standard for indirect measurement of cardiorespiratory performance (Zhang et al. 2017) and the practicability as well as the motivation of the participants was very high for the selected test. Although all participants can be seen as rather fit in comparison to norm values reported in literature (Bohannon 2007) we found differences between fit and less fit in line with dedifferentiation. When measurements took place, participants had either learned golf within the last 22 weeks or continued with their normal daily activities. The golf training could have obviously influenced the results. In this study we were more interested in the long-term effects associated with cardiorespiratory fitness, reflected by the 6-min walking test, than in short-term effects caused by golf training. Moreover, Voss et al. (2016) pointed out that the influence of cardiorespiratory fitness and short-term physical activity on brain networks are independent phenomena. Of course, longitudinal recordings and a more objective measurement of cardiorespiratory fitness and daily activity would extend presented findings. As this study was part of a bigger intervention study, sample size was fixed a priori. The primary outcome of this study was the ADAS-Cog based on the randomized controlled trial of Lautenschlager et al. (2008). The standardized mean difference for ADAS-Cog was -1.22. The drop-out rate was set to 20%. It was estimated that a sample size of 46 participants (23 in each group) would provide 95% power for detecting a significant group difference. A healthy young control group would be beneficial in order to categorize our findings.

## Conclusion

In applying DMD to continuous EEG recordings during rest and three different tasks, we considered both topological properties and the temporal dynamics of task-related brain networks. Thus, we identified electrophysiological signatures of age-related brain reorganization processes in fit and less fit older adults. Fit participants showed higher task specificity, i.e., more differentiated brain activation patterns, as well as higher prominence of these patterns, indicating less neural noise throughout task execution. Our findings support the idea that physical fitness manifests in task-related brain network activation patterns that are in line with reduced dedifferentiation in older adults.

## Supplementary information

Below is the link to the electronic supplementary material.

Supplementary material 1 (XLSX 314 kb)

## Data Availability

Data and material are available from the corresponding author, SV, upon reasonable request.
